# Attitudes to participating in a birth cohort study, views from a multiethnic population: a qualitative study using focus groups

**DOI:** 10.1111/hex.12445

**Published:** 2016-06-17

**Authors:** Neeru Garg, Thomas P. Round, Gavin Daker‐White, Peter Bower, Chris J. Griffiths

**Affiliations:** ^1^ NIHR School for Primary Care ResearchCentre for Primary CareUniversity of ManchesterManchesterUK; ^2^ Department of Primary Care and Public Health ResearchKing's College LondonLondonUK; ^3^ NIHR Greater Manchester Primary Care Patient Safety Translational Research CentreUniversity of ManchesterManchesterUK; ^4^ NIHR School for Primary Care ResearchManchester Academic Health Science CentreUniversity of ManchesterManchesterUK; ^5^ Centre for Health SciencesQueen Mary University of LondonLondonUK

**Keywords:** birth cohort, children, ethnicity, focus groups, recruitment, research participation

## Abstract

**Background:**

Recruitment to birth cohort studies is a challenge. Few studies have addressed the attitudes of women about taking part in birth cohort studies particularly those from ethnic minority groups.

**Objective:**

To seek the views of people from diverse ethnic backgrounds about participation in a proposed birth cohort examining the impact of infections.

**Design and setting:**

Eight focus groups of pregnant women and mothers of young children took place in GP surgeries and community centres in an ethnically diverse area of east London. Purposeful sampling and language support ensured representation of people from ethnic minority groups. Audio recordings were taken and transcripts were analysed using the Framework approach.

**Main outcome measures:**

The views of participants about taking part in the proposed birth cohort study, in particular concerning incentives to taking part, disincentives and attitudes to consenting children.

**Results:**

There was more convergence of opinion than divergence across groups. Altruism, perceived health gains of participating and financial rewards were motivating factors for most women. Worries about causing harm to their child, inconvenience, time pressure and blood sample taking as well as a perceived lack of health gains were disincentives to most. Mistrust of researchers did not appear to be a significant barrier. The study indicates that ethnicity and other demographic factors influence attitudes to participation.

**Conclusions:**

To recruit better, birth cohort studies should incorporate financial and health gains as rewards for participation, promote the altruistic goals of research, give assurances regarding the safety of the participating children and sensitive data, avoid discomfort and maximize convenience. Ethnicity influences attitudes to participation in many ways, and researchers should explore these factors in their target population.

## Introduction

The difficulties in recruiting sufficient numbers of participants to large research studies are widely acknowledged.^1^, ^2^ Birth cohort studies are important epidemiological research tools providing longitudinal observational data. In order to achieve scientific validity and to guard against typically high attrition rates, they need to recruit very large numbers of participants. A very few studies have addressed the attitudes of eligible women to participating in birth cohort studies.^3^, ^4^

Nechuta *et al*. used questionnaires to survey the opinions of pregnant women towards taking part in research involving interviews and biological sample taking.^5^, ^6^ They showed that 24% of participants would be unwilling to provide biological samples and 9–34% were unwilling to take part in non‐invasive research procedures such as interviews irrespective of compensation. Unwillingness to participate was linked to a higher educational status. These studies collected numerical data and thus could not explain these findings. The wider literature addressing factors which motivate people to take part in all other types of research suggests that participants are motivated by altruism,^7^, ^8^, ^9^, ^10^ an expected personal gain in health‐related knowledge,^11^ perceived additional health benefits of taking part^12^ and payments.^13^ Disincentives are found to be a lack of trust in the health profession/research staff,^7^ including concerns about data confidentiality, inconvenience or discomfort, lack of information about the study^13^ and a reluctance to take part in higher risk studies.

Parents provide consent to take part in research on behalf of their children. Literature addressing attitudes of parents towards consenting children to participate in epidemiological research is limited as most studies focus on clinical trial participation.^12^, ^14^, ^15^ In addition to altruism,^12^ parents are motivated by direct health benefits to their child of inclusion but are concerned about exposing their child to harm and experience significant conflict when making the decision.^14^, ^15^ There may be greater difficulty in recruiting children to epidemiological studies that are observational, and so generally recruit healthy children, rather than those studies which recruit unwell children,^16^ probably due to the perceived lack of direct benefit to the child's health from participating in the former case. Socio‐economic class may also be important where lower socio‐economic class has been linked to greater participation.^17^

People from ethnic minority groups are underrepresented in research including birth cohort studies.^18^, ^19^, ^20^, ^21^ The Millennium cohort study which was a large recent national British birth cohort study also showed that people from ethnic minority groups had a higher attrition rate than those from non‐minority groups.^22^

The underrepresentation of people from ethnic minority backgrounds in clinical research has important ethical and scientific implications. Lack of representation of people from ethnic minority groups results in studies lacking generalizability to the population as a whole, and differences in metabolism, pharmacodynamics and pharmacokinetics between people from different ethnicities might mean that people from ethnic minority groups do not benefit from best treatments.^23^ Most studies addressing the opinions of ethnic minority groups about taking part in research have occurred in the USA, and there is a paucity of such studies in the UK. The US studies highlight mistrust of research and health professionals as a significant disincentive to research participation, particularly with respect to those with African American ethnicity.^24^, ^25^, ^26^, ^27^ Explanations for this may be related to the ‘legacy of exploitation’ in the social history of this group^28^ and the significant damage to the relationship between government researchers and African Americans caused by the Tuskegee U.S. public health service Syphilis study.^29^ Further determining factors for participation of people with ethnic minority backgrounds in the US literature were a lack of access to clear information in the appropriate language, inconvenience, stigma of inclusion in research, concerns regarding legal status and concerns regarding health insurance.^9^ These findings appear congruent with two UK studies on this subject,^18^, ^30^ but caution must be applied when generalizing US findings to the UK which has an entirely different ethnic composition, social history and health system.

In order to improve the quality of birth cohort studies, there is a need to understand the factors which motivate women to take part as well as those which might act as disincentives and to include the views of people from ethnic minority groups.

### Study aims

The aim of this study was to explore the attitudes of women around regarding themselves and their children to taking part in a large proposed birth cohort study.

The proposed birth cohort study, which at the time of writing is awaiting funding approval, will address as its main subject the relationship of infection in early life to health outcomes in later life. It will collect information, measurements and biological samples from women during pregnancy and from their offspring who will be followed up into adulthood. Samples will include swabs, urine samples, umbilical cord samples and blood samples, thus allowing parameters such as immunity, genetics, biochemistry amongst others to be studied.

The proposed birth cohort study will take place in two inner city boroughs of East London: Hackney and Tower Hamlets. This area is one of the most ethnically diverse in the UK (Table 1). This study actively sought to include the views of people from several ethnic minority backgrounds.

**Table 1:** 2011 Census data, Office for National Statistics

	UK	Hackney	Tower Hamlets
Total population	63 182 178	246 270	254 096
% White	87.1	54.5	45.1
%Gypsy/Traveller/Irish traveller	0.1	0.2	0.1
% Mixed/Multiple ethnic groups	2.0	6.4	4.1
% Asian/Asian British: Indian	2.3	3.1	2.7
% Asian/Asian British: Pakistani	1.9	0.8	1.0
% Asian/British Asian: Bangladeshi	0.7	2.5	32.0
% Asian/British Asian: Chinese	0.7	1.4	3.2
% Asian/British Asian: Other Asian	1.4	2.7	2.3
%Black/African/Caribbean/Black British	3.0	23.1	7.3
% Other ethnic group	1.9	5.3	2.3

### Methodology

We chose to use focus groups to explore this issue. Focus group design enables participants to answer questions individually as well as having the opportunity to interact with each other.^31^ They are therefore useful in exploring attitudes as they allow participants to expand on and clarify view points in the context of other group members’ contributions.^32^ A further advantage is that focus groups provide a ‘social context’ to the information gathering.^32^ In this way, focus groups might more closely reflect the manner in which decisions about participation in a large birth cohort study naturally take place in the community, that is a mix of individual and shared perspectives. For these reasons, focus groups were felt to be the most appropriate and efficient method of data collection to achieve the study aims.

Participants were recruited by researchers from waiting rooms of five different General Practices and two community centres in Hackney and Tower Hamlets. All potential participants were women who were either pregnant or had young children under the age of 5 years. These centres were chosen because they were situated in areas of differing ethnic composition, thus aiding recruitment of participants from diverse ethnic backgrounds. Ethics approval was obtained prior to contact with participants^33^ and those recruited gave written informed consent.

The participants were then organized into focus groups from the same, self‐reported, ethnic background. This meant that participants shared the same first language and were thus able to communicate with each other effectively during the focus group discussions. This format was also more convenient for most participants as those sharing the same ethnic background tended to reside in the same geographical area and hence preferred the same focus group venues. The resulting ethnic homogeneity within groups provided an opportunity to make observations about the degree of sharing of themes between people from the same ethnicity. It is important to note that such inferences were significantly limited as each individual group represented a small convenience sample, and thus, observations could not be generalized to an ethnic group as a whole. This limitation is discussed later when discussing the strengths and limitations of this paper.

Demographic data was captured via a questionnaire regarding the level of education, employment status, size of family, income and medical history. An additional question was completed at the end of the discussions asking whether the participant would take part in the proposed birth cohort study should it go ahead.

One of three different researchers facilitated the groups. They first described verbally to the participants the details of the proposed birth cohort study. An interpreter was required for this in three groups (Turkish, Bangladeshi, Chinese). Presenting the information to participants on the day ensured responses were fresh and unrehearsed. After checking understanding, the groups were then encouraged to discuss the study using a topic guide (Table 2). Facilitator involvement in discussions was minimal with facilitators adopting a structured eavesdropping approach.^34^

**Table 2:** Topic guide

Topics
1. What do you make of this study; do you understand what it is about?
2. Do you think this study is relevant to you or your child Do you think it might be useful to you or your child in the future?
3. Is this study acceptable to you? Would you be happy to do all of the things the study asks of you? Please consider the following: Testing in pregnancy. Providing of sputum. Providing of cheek cells, faeces, vaginal swabs, skin swabs, throat swabs. Giving several blood samples, and your child giving several blood samples. Using the father's data
4. Do you think that this study is a good idea? Would you take part? Would you tell your friends about it?
5. What puts you off from doing the study?
6. Is there anything we could find out for you when we do this study?
7. What do you think would be the best way to get others involved?
8. How long would you and your child be happy to continue to engage with the study?
9. If you took part what are your reasons for doing so? Why might you take part in the study? What is good about this study?

All conversations were digitally recorded and transcribed verbatim. Where necessary, interpreters translated scripts into English for analysis purposes. The decision to stop further data collection was made when it was felt that few new themes were emerging.

### Analysis

Data was thematically analysed using the Framework approach^35^ A distinctive aspect of the approach is that it allows themes to develop both from the research questions and from the narratives of research participants.^36^ This approach has primarily been used in health‐care settings.^37^

The Framework approach involves a five‐step process:

Familiarization – immersion in the raw data.
Identifying a thematic framework – identifying all the key issues, concepts and themes by which the data can be examined and referenced.
Indexing – applying the thematic framework or index systematically to all the data in textual form by annotating the transcripts with numerical codes.
Charting – rearranging the data according to the appropriate part of the thematic framework to which they relate, and forming charts.
Mapping and interpretation – using the charts to define concepts and find associations between themes with a view to providing explanations for the findings.^38^

Analysis was carried out by two of the three researchers who facilitated the group discussions who applied the Framework approach to the focus group transcripts.

Transcripts were imported into the data‐handling programme MAXQDA 10,^39^ which was used to facilitate indexing, charting and mapping. Codes were refined and organized into a thematic framework with regular validation of themes between the other members of the research team which can improve consistency and reliability.^38^

## Results

A total of forty women took part in eight focus groups which took place between July 2009 and January 2010. Their demographic details are shown in Table 3. The main reason given by those who refused to participate was a lack of time/inconvenience. The possibility of bias in the sample towards those who were either unemployed or had fewer children that this introduces was not found in the demographic data (Table 3). Discussions yielded recordings within all eight groups lasting between sixty and one hundred and twenty minutes. The identified themes are divided into the following themes and subthemes.

**Table 3:** Questionnaire data – participant characteristics and exit poll

	White British	Black British	African and Caribbean	Bangladeshi	Turkish	Chinese	Jewish
No of focus groups	2	1	1	1	1	1	1
Participants	8	4	6	7	4	5	6
Age range	25–42	22–31	22–42	21–39	24–30	24–39	20–47
UK born	7	4	0	0	0	0	0
Fluency in English							
Fluent	8	4	2	0	1	0	6
Intermediate	0	0	4	1	0	0	0
Not	0	0	0	6	1	5	0
Currently working	5		1	0	1	0	3
Age finished education							
<16	0	0	0	4	1	0	0
16–18	6	2	2	2	0	5	5
19–22	2	1	2	1	1	0	0
>22	0	1	2	0	0	0	1
Household income							
Not stated	0	2	4	1	0	0	2
<£20000	1	2	0	4	2	5	4
20–40 000	4	0	2	1	0	0	0
40–60 000	1	0	0	1	0	0	0
>60 000	2	0	0	0	0	0	0
Number of children							
0	0	0	1	0	1	0	0
1	4	3	1	2	1	1	1
2	1	1	2	0	0	1	2
3+	3	0	2	5	0	3	3
Would take part in the proposed birth cohort?	6 (80%)	4 (100%)	5 (83%)	7 (100%)	2 (50%)	5 (100%)	4 (67%)

1. Incentives for research participation
  - Altruism
  - Perceived non‐material benefits
  - Material incentives
2. Disincentives
  - Lack of personal benefits
  - Mistrust
  - Conflict about consenting their child
  - Potential for psychological harm
3. Attitudes towards practical/design aspects
  - Sampling
  - Convenience

(1a) Participants were motivated by altruism to add to scientific knowledge and help future generations.

At the end of this as long as you come out with some good theories, some good research to say these infections are more common in this or that case then that's fine. We want to know more about our kids and the greater good of people. (African and Caribbean group)

Not all participants, however, expressed altruism in terms of helping ‘science’ or the general population but rather in terms of helping specific groups such as future generations of their own family or ethnic groups to which they belong or have an affinity with.

If it doesn't help my kids it would help my grandchildren. (White British group)

Part of my reasons for taking part I hope one day, maybe the study would go back to Africa and be able to help mothers who really need it. That's one of the major reasons why I would participate. (African and Caribbean group)

(1b) Some participants thought that they and their children would receive superior health care than others if they took part in the study by means of regular contact with researchers and an increased frequency of sample taking.

Having the opportunity to have someone with a one to one like monitoring your child whilst other parents won't have that opportunity because they don't want to take part or don't know about it. (Black British group)

‘….I've got you guys testing her urine and this, that and the other, it sort of reassures me there is nothing wrong with her and she's normal. (Black British group)

Note that such additional health benefits were not described to the groups; none were inherent in the design of the proposed birth cohort study.

(1c) Participant wanted some form of reimbursement for participation. The preferred strategy varied considerably according to individuals and not all found a cash payment acceptable.

its also more polite to use vouchers. I had one man, one time, give money for a survey and he started taking out of a bag £1, £2…I felt like such a beggar (Jewish group)

Other suggested forms of reimbursement were cash, school vouchers and movie tokens for children.

Groups did not in general discuss the magnitude of payment.

(2a) In contrast to the point made in the previous section describing incentives, some participants felt the birth cohort was unlikely to yield any personal health gains.

It sounds like the children taking part in this might not particularly benefit from it because you are just collecting information aren't you, you cant make any diagnosis. (White British group)

Some participants perceived a lack of benefit of involving their child in research when their child was healthy.

From a cultural background….its like ‘why do you have to? My child is healthy, there's nothing wrong with me’, I'm not interested in infection if my child is healthy (African and Caribbean group)

(2b) There were a plethora of stories regarding bad experiences perceived to be caused by health professionals.

I had a friend who was even, on the second baby, had a caesarean, and it stopped her from having more babies, without her consent. (African and Caribbean group)

Despite this, participants did not, in contrast to US studies of certain minority groups,^24^ express high levels of distrust regarding the intention of researchers.

However, the notion that their child was being used as a ‘guinea‐pig’ was mentioned in several groups. This might belie in these groups an underlying mistrust of the researchers and view of them as exploiting their child.

I am not sure I think it's a good thing but at the same time it's a bit intrusive, I am not sure…its like she's a guinea pig or something. (Black British group)

And there was a widespread concern about the safe handling of confidential information.

I think it is a lot to ask of people and if people are willing to give that commitment we have to be sure of the security aspects as well, (White British group)

(2c) Mothers demonstrated discomfort when making the decision on behalf of their child and were often conflicted between the potential benefits and risks of the study when considering enrolling their child.

It's good to get information like you said, and I am not sure I would want my child to be a guinea pig. Me's different you know because I am volunteering myself but for me to volunteer for her (Black British)

(2d) Some, the Turkish group in particular, voiced concerns that the child could be ‘singled out’ through their involvement in the study when other children were not and that this could be potentially psychologically harmful.

My son [is] two and half years old and I am worried about whenever he start to think he can say, “Why me and not the others, why not my friends?” (Turkish group)

(3a) Taking samples was discussed in all groups with a general consensus that non‐invasive swabs were better and blood sampling, due to the discomfort of needle use, was unacceptable to most.

Swabs is better (than blood sampling), people wouldn't mind testing, (White British group)

Not the blood tests obviously because they don't like needles (White British group)

Some religious/cultural references emerged when discussing blood taking in some groups notably the spiritual connection to body samples in the African and Caribbean group, and views around testing for Down's syndrome in the Jewish/British Jewish group.

Because where I come from spiritual meanings come right into it, so when you're talking about samples or even telling people personally about it, some people might not be cool about it (African and Caribbean group)

It all depends what they are. As for example most Jewish women would not test for Down's syndrome, we would not have an abortion anyway. (British Jewish group)

(3b) Time pressure was a significant disincentive; however, it was suggested that tying in data collection to routine visits and holding the study locally could mitigate this effect.

When you're having your smear test or something you could have your swab test done at the same time or if things were tied into what was happening anyway (White British group)

The doctor's surgery was put forward as an acceptable location due to its locality and the familiarity with the health professional.

I think it's easier at your GP because it is local to where you live and you are used to seeing certain doctors better than going to the hospital as far away. (Black British group)

Eighty‐three percent of participants expressed in the anonymous exit poll that they would be willing to take part in the proposed birth cohort should it go ahead. (Table 3). This finding might predict a high participation rate in the birth cohort study; however, the members of women, as the focus groups and as such already taking part in research, may represent a group positively biased towards research participation than the rest of the target population. This and the fact that the responses were for the birth cohort study presented hypothetically may mean that this finding is not predictive of actual levels of recruitment.

Table 4 summarizes the main factors and describes where these factors showed convergence in the sample and where opinion was divided or expressed more strongly by certain individuals or groups.

**Table 4:** Main incentives and disincentives: and if represent convergence of opinion or views held by single participants or certain groups

	Convergence of opinion	Views held by single participants or certain groups
Incentives	Altruistic motivation. Importance in gaining knowledge about immunity and infection. Material incentives. Health benefits from participation are helpful Study should be held locally. Engagement of children.	Altruistically motivated to help certain groups. Type and magnitude of material incentive. Perceived presence of health benefits.
Disincentives	Concerns about data protection. Use of needles and blood tests. Conflict when consenting a child for research. Consenting healthy children Time pressure. Intrusiveness Language barriers	Perceived lack of presence of health benefits. Mistrust of researchers Risk of psychological harm to children. Religious and cultural beliefs around sampling.

## Strengths and limitations

### Strengths

To our knowledge, this is one of only very few studies which addresses the factors which motivate participants to take part in a birth cohort study and is the only one to do so using focus group methodology. It is also one of very few studies to include the views of people from ethnic minority groups in the UK and attitudes towards consenting children for epidemiological research.

### Limitations

The literature suggests that attitudes to birth cohort study participation may be influenced by socio‐economic status and other factors such as education level. Individual demographic data was collected; however, individual responses within group discussions were not and so it was not possible in this study to link individual responses to demographic factors. Due to time and resource limitations, it was not possible for the non‐English‐speaking group recordings to be independently translated and transcribed separately to the health advocates used in the focus groups introducing the possibility of bias, or to perform respondent validation.

As stated, the study included the opinions from people from ethnic minority groups who are often underrepresented in research. Each individual focus group, however, in itself represented a small convenience sample, and therefore, this study cannot draw conclusions regarding the entire ethnic group as a whole. Neither did the analysis compare all themes across all groups. However, the study is able to detect and describe instances where certain beliefs appear to be held more strongly by one group/s over the others. The significance of this is described with caution and with the study's limitations in mind.

The presence of a researcher in the room could have affected responses, particularly when considering the topic of discussion was attitudes to taking part in research. Minimal intervention from researchers, who adopted a structured eavesdropping approach, assurances of confidentiality and use of topic guides minimized this effect.

## Discussion

### Incentives: Altruism

In agreement with the literature,^7^, ^8^ we did find that altruism was found to be a motivating factor encouraging many to participate. There was a sense that a study about infection and immunity was important and a desire was expressed across all groups to further advance scientific knowledge in this area and help others. Some referred to helping society as a whole, the ‘greater good’, whereas others wanted to help groups that they were affiliated to such as future generations of their own family or people from the same ethnic background living either within or outside of the UK. This finding substantiates other findings in the literature where participants have been more motivated to participate in research if felt that this would help underrepresented groups of people with ethnic backgrounds similar to their own.^18^

Certainly, altruistic motivations occur in different forms^10^ and recruitment strategies should acknowledge this variation in order to be maximally effective. It was unclear to what extent altruistic notions would motivate participants to take part in the birth cohort. Some argue that the importance of altruism is overrated and is unlikely to be the predominant factor.^10^ Other factors are considered below.

### Non‐material incentives

There was the perception that participation in the birth cohort study would confer some additional health gains. Participants assumed that as a consequence of the processes of the birth cohort study, they would receive superior health care in relation to those not taking part. This was a concept that was very appealing to participants and acted as an incentive, which is consistent with the literature that states that perceived health gains are an important motivational factor in participating in clinical research.^8^

Participants believed that these gains would be achieved through having more time with doctors/researchers, regular routine sample taking (conferring additional ‘protection’ or ‘monitoring’ of their child) and a gain in health‐related knowledge. The appeal of increased time with doctors may reflect the inherent restrictions on the availability of health professionals in the health‐care system. Routine sample taking would be unlikely to provide a large health benefit as it is widely acknowledged that routine samples taken in the absence of clinical indications/symptoms yield few health gains^40^ It is, however, conceivable that receiving normal results may alleviate parental anxiety which could in itself constitute a type of health gain.

These over‐exaggerations about the benefits in health‐care terms of the research process were relatively more prevalent in the non‐English‐speaking groups. English‐speaking groups were more likely to assume that the birth cohort study would not yield any significant direct health benefits in a time frame relevant to them or their family. Further, we also found that non‐English‐speaking groups tended to have a worse understanding of the concepts regarding illness and disease. The reasons for this were not clear and could not obviously be attributed to differences in levels of educational attainment according to the demographic data (see Table 3). Other explanations could be cultural differences in thinking about health and illness or the possibility that language barriers might inhibit adult learning. Such misconstructions about the benefits of a birth cohort study could contribute to higher attrition rates – when the expected benefits do not materialize during the course of the study although this was not shown and recruiting under false pretexts is exploitative. Care should be taken, particularly in non‐English‐speaking groups, to explain the study cohort clearly, set expectations and to explain with language support where necessary the risks and benefits of the study.

### Material incentives

In agreement with the literature that material incentives do improve participation rates, participants were keen for some form of material reward.^41^ There is an on‐going ethical debate about the use of material incentives to improve research participation, particularly the concern that their use may lead to participants ignoring the risks involved in studies.^41^ It is thought that money might be a greater incentive amongst economically disadvantaged groups who may therefore be more susceptible to this form of exploitation.^42^ The focus groups were not very forthcoming in discussions regarding financial incentives, possibly due to the sensitive nature of the topic and so these findings were not corroborated. Differences were noted, however, in the preferred forms of reimbursement. Some preferred non‐cash reimbursements viewing cash reimbursements as distasteful.

Further, the point was made that it would be important to engage the children, not just provide a financial incentive to the parents, by making the study fun and this could be done by possibly involving schools.

### Disincentives: Trust and data confidentiality

Mistrust of researchers and research processes is quoted in the literature as a significant disincentive to participation amongst some ethnic minority groups in the USA.^26^ There was less evidence for this kind of mistrust of research in this study. This might reflect the differences in the social history of ethnic minority groups in the two countries as well as the absence in the UK of high profile cases of mistreatment of ethnic minority groups in research such as the Tuskegee study.^29^ It could also reflect more equitable access for people from ethnic minority groups in the public health service of the UK as opposed to the private health system of the USA although this was not shown. An indication that a level of mistrust towards researchers/research processes might exist was suggested by the attitude held by some participants that their child was being used as a ‘guinea‐pig’. This is a term often seen in the US literature discussing attitudes of African Americans to research.^43^ It alludes to a sense of exploitation and may mean that mothers lack trust in the researchers to fully protect their child from harm during the study.

Many participants were concerned about the ability of the researchers to maintain confidentiality of sensitive data, particularly with regard to their children and would need reassurance about the safe keeping of data in order to participate. The GP surgery records were perceived to be a secure place to keep data.

### Disincentives: Consenting on behalf of children for epidemiological research

Congruent with the literature, participants displayed a cautious attitude to enrolling their children in research and considered carefully any potential risks posed from involvement.^14^, ^15^ The main perceived risks associated with the birth cohort study were discomfort from painful procedures particularly blood tests, the potential for loss of sensitive data and the potential for psychological harm to their child. The latter factor was felt strongly in the Turkish group where concern was expressed around their child being ‘singled out as different from other children’ by participating and a general concern about the effect of ‘the intrusion’ of researchers into their lives. Once again it is not possible from the small convenience sample to extrapolate this view to all participants with Turkish ethnicity; however, the significant presence here merits a more detailed exploration.

The findings in the literature that it may be harder to motivate parents to enrol well children into observational research studies, such as a birth cohort study rather than clinical trials, where there is a treatment benefit were well supported in the data.^16^, ^24^

### Practical aspects: Sampling and inconvenience

In disagreement with the questionnaire studies^6^ described in the introduction, blood sampling was generally found to be unacceptable in all groups. There was an association found between previous bad experiences with needles, often during painful vaccinations and negative perceptions of blood tests.

Religious and cultural influences were also seen in relation to blood sample taking. In the African Caribbean group, one participant described the belief that in her culture, blood was connected to the concept of the ‘spirit’ or ‘life’, making blood sample taking unacceptable. In the Jewish group, some participants would not permit blood taking if this was for genetic testing. This was linked to prohibitive religious laws surrounding abortion in some denominations of Judaism.^44^

The proposed birth cohort study was perceived to be potentially time‐consuming and inconvenient both factors which were strong disincentives to participation. Data indicated that tying the data collection into routine visits to doctors could mitigate these factors as could holding the study local to the patient's home. The GP surgery was again identified as an acceptable location.

## Conclusions

This qualitative study uniquely explored as its main aim attitudes to participation in a birth cohort study using focus groups methods. To our knowledge, it is the only study thus far to do so and thus adds significantly to the understanding in this area. The study produced rich data and identified several factors which incentivized women to participate in a birth cohort study and those which acted as disincentives.

Views were effectively obtained from participants from diverse ethnic backgrounds. Small convenience samples meant that interpretation was limited, but instances where certain beliefs appear to be held more strongly by one group/s over the others were reported. Findings suggested that people from differing ethnic backgrounds might differ in terms of the type of perceived risks to their children, altruistic motivations, level of health literacy possibly as a function of language barriers and this could in turn result in differing perceptions of gains from the study. There was some limited evidence of differences in attitudes to biological sample taking between groups of different ethnicities. Consideration of the impact this might have on research recruitment, particularly of people belonging to ethnic minority groups, merits further study.

### Implications for policy

Our study suggests that to improve recruitment to birth cohort studies, researchers should provide a form of material incentive and provide a form of non‐material ‘health benefit’ through participation. They should make efforts to make the study engaging for children possibly by involving schools, provide reassurances to parents about data protection, minimize the use of needles, reduce intrusiveness and extra time needed for the study possibly by tying it into naturally occurring visits to health professionals and collect data using local locations such as at the GP surgery.

Researchers should consider carefully the demographic composition of their target population due to the potential influence of socio‐economic status, language and ethnicity on the several important factors determining research participation.

## Sources of funding

Bart's Charity. The paper was supported in part by the UK National Institute of Health Research (NIHR) School for Primary Care Research. The views expressed in this publication are those of the authors and not necessarily those of the NHS, NIHR, or Department of Health.

## Conflict of interest

No Conflict of interests have been declared.
